# Trends in urine lead and associated mortality in US adults: NHANES 1999–2018

**DOI:** 10.3389/fnut.2024.1411206

**Published:** 2024-05-30

**Authors:** Qiong Wang, Jing Wu, Xiaoqun Dong, Wenquan Niu

**Affiliations:** ^1^Graduate School, Beijing University of Chinese Medicine, Beijing, China; ^2^Department of Pediatrics, China-Japan Friendship Hospital, Beijing, China; ^3^Center for Evidence-Based Medicine, Capital Institute of Pediatrics, Beijing, China; ^4^Department of Medicine, The Warren Alpert Medical School, Brown University, Providence, RI, United States

**Keywords:** urine lead, trend, all-cause mortality, cardiovascular disease-specific mortality, cancer-specific mortality

## Abstract

**Objectives:**

This study aimed to describe the trends of urine lead among US adults aged ≥45 years and to explore its association with all-cause and disease-specific mortality.

**Methods:**

This study enrolled 9,669 participants from the National Health and Nutrition Examination Survey, 1999–2018. Trends in urine lead were described by logistic regression analysis using the survey cycle as a continuous variable. Cox proportional hazard regression analyses were used to quantify the association between urine lead and mortality.

**Results:**

There was an obvious decline in urine lead concentrations from 1.203 μg/L (95% confidence interval [CI]: 1.083–1.322) in 1999–2000 to 0.478 μg/L (95% CI: 0.433–0.523) in 2017–2018, and this decline was statistically significant (*P* < 0.001). Referring to the first tertile of urine lead concentrations, risk magnitude for all-cause mortality was significantly and linearly increased after adjustment (*P* = 0.026 and 0.020 for partially and fully adjusted models, respectively), and significance was attained for the comparison of the third vs. first tertile after full adjustment (hazard ratio [HR]: 1.17, 95% CI: 1.01 to 1.35). Treating urine lead continuously, the risk for all-cause mortality was statistically significant (HR: 1.18 and 1.19, 95% CI: 1.01 to 1.39 and 1.00 to 1.40 for partially and fully adjusted models). For cardiovascular disease-specific and cancer-specific mortality, there was no hint of statistical significance.

**Conclusions:**

Our findings indicated that urine lead exhibited a declining trend from 1999–2000 to 2017–2018 in US adults aged ≥45 years, and high urine lead was a significant and independent risk factor for all-cause mortality.

## Introduction

Lead is a highly toxic chemical that persists in housing, soil, water, and consumer products, and it can accumulate in the body over time. Body lead concentrations have declined significantly over the past few decades (1, 2), whereas lead poisoning continues to be a serious public health concern (3, 4), causing a loss of 21.7 million disability-adjusted life years (DALYs) globally, as recorded by the World Health Organization. In 2019, the number of stroke deaths worldwide due to lead exposure exceeded 300,000 (5). In the United States, lead exposure is responsible for approximately 400,000 deaths every year (6). Epidemiological and toxicological studies have shown that lead exposure can trigger the occurrence and progression of many adverse consequences, such as learning and behavior disorders, impaired kidney function, cardiovascular events, decreased fertility, and cancer, especially in older individuals (7, 8).

Numerous studies have shown that older individuals are at a high risk for recurring endogenous exposure to lead, which accumulates over time in the skeleton (9, 10). Generally, environmental lead absorbed into the bloodstream has a half-life of 30 days, and when lead binds to circulating erythrocytes and is deposited in bones, its half-life can extend to 20–30 years. With aging, body lead concentrations increase due to the increase in bone demineralization and the release of stored lead (11). The previously mentioned lines of evidence collectively demonstrate the long-acting impact of lead exposure on human health (12). However, the evidence base for the long-term consequences of lead exposure is sparse in older individuals. A better understanding of the population-based characteristics of body lead and its associations with mortality will provide more insights into the pathogenicity of lead and will facilitate the development of preventive strategies.

To yield more information, we aimed to describe the temporal trends of urine lead from 1999–2000 to 2017−2018 among US adults aged ≥45 years and to explore the association of urine lead with all-cause and disease-specific mortality.

## Methods

### Analytical dataset

All data have been made publicly available by the National Center for Health Statistics and can be accessed/downloaded at the website https://wwwn.cdc.gov/nchs/nhanes/Default.aspx.

### Study participants

Study participants were enrolled in the National Health and Nutrition Examination Survey (NHANES), 1999–2018. Since 1999, the NHANES has been a continuous, multistage, nationally representative survey of non-institutionalized adults and children in the United States. Data from each participant were gleaned through household interviews and physical examinations conducted at a mobile examination center and released in 2-year cycles.

In this study, adults aged 45 years or more who were not pregnant and had complete urine blood data at enrollment were eligible for inclusion from 10 cycles between 1999–2000 and 2017–2018. There were 9,669 adults in the final analysis.

### Urine lead

Urine specimens were collected during medical examinations. After confirming the absence of background contamination in the collected materials, the NHANES research team collected a urine specimen from each respondent on site. The urine specimens were frozen, stored, and shipped to the Division of Laboratory Science at the U.S. Centers for Disease Control and Prevention in Atlanta, Georgia, for mass analysis. Inductively coupled plasma mass spectrometry (ICP-MS) was used to determine the concentrations of lead in urine. If urine lead content was below the limit of detection (LOD), its concentration was calculated as the LOD divided by the square root of two. Considering the fact that creatinine is an established marker of kidney damage, lead concentrations were standardized by urine creatinine.

### Covariates

Several sociodemographic variables were extracted from the NHANES dataset, including age, gender, race and ethnicity, body weight, and height. Age was divided into two groups at the cutoff of 65 years. Race and ethnicity data were collected by trained interviewers using fixed categories from the National Center for Health Statistics. The body mass index (BMI) was calculated as weight in kilograms divided by height in meters squared and was categorized as underweight or normal weight, overweight, and obesity based on the World Health Organization criteria.

### Mortality

The NHANES Linked Mortality Files include the continuous NHANES years (1999–2018) and provide mortality follow-up data from the date of survey participation through 31 December 2019. The underlying causes of death were recorded according to the ICD-10 codes: cardiovascular diseases, including heart diseases (ICD-10 codes I00-I78), and malignant neoplasms (ICD-10 codes C00-C97). Survival time (in months) was defined as the time from the date of participation to the date of death or the date of the last follow-up on 31 December 2019, where applicable. In this study, all-cause mortality, cardiovascular disease (CVD)-specific mortality, and cancer-specific mortality were examined.

### Statistical analyses

Survey analysis procedures were adopted to account for sampling weights, stratification, and clustering in the NHANES complex sampling design to derive nationally representative sample estimates. Due to deviations from normal distributions, urine lead concentrations were ln-transformed. Age-standardized and weighted concentrations of urine lead with 95% confidence intervals (CIs) were calculated. Temporal trends of urine lead were examined by logistic regression analysis using the survey cycle as a continuous variable and by the Mann–Kendall trend test.

The proportional hazards assumption of the Cox proportional hazards models was tested using Schoenfeld residuals, and no obvious deviation from proportionality in hazards over time was observed (*P* > 0.05). Then, Cox proportional hazards regression analyses were adopted to explore the association of urine lead on both continuous and categorical variable scales with all-cause and disease-specific mortality before and after adjusting for covariates, including age, gender, race and ethnicity, and BMI category. In addition to the overall estimates, subgroup analyses were also performed, and the interaction between subsidiary estimates was examined by the Z test proposed by Altman and Bland (13).

The dose–response relationship between urine lead and mortality outcomes was examined in two steps. First, the multivariate-adjusted relative risk across the tertiles of urine lead concentrations was tested. Second, the three-knot restricted cubic spline was fitted to visualize the shape of the correlation between urine lead and all-cause and disease-specific mortality by modeling the log-transformed lead concentrations in urine (14). The dose–response relationship was assessed by the Wald test (15).

All analyses were completed using the STATA software version 14 (Stata Crop LLC) and the R coding platform version 4.3.1. A two-sided *p*-value of < 0.05 is indicative of statistical significance.

## Results

### Characteristics of study participants

The baseline characteristics of study participants are presented in Table 1. For 9,669 eligible adults aged ≥45 years with complete urine lead and mortality data, the mean (standard deviation [SD]) age was 62.8 (0.11) years, and 4,836 (50.02%) of the participants were women. Survey-weighted percentages of non-Hispanic white, non-Hispanic Black, and others were 47.25%, 20.77%, and 31.98%, respectively.

**Table 1:** Baseline characteristics of study participants aged ≥45 years in the National Health and Nutrition Examination Survey (NHANES), 1999–2018^a^.

**Characteristics groups**	**1999–2000**	**2001–2002**	**2003–2004**	**2005–2006**	**2007–2008**	**2009–2010**	**2011–2012**	**2013–2014**	**2015–2016**	**2017–2018**
Sample size	802	857	899	816	1,114	1,139	944	1,006	1,017	1,075
Follow-up, mon	178	168	153	140	140	127	90	70	47	24
Age, years	64	63	65	63	62	63	62	62	62	63
< 65	68.78 (65.86, 71.55)	70.35 (66.24, 74.15)	68.01 (62.82, 72.80)	70.13 (65.78, 74.15)	72.68 (68.39, 76.58)	69.34 (64.92, 73.44)	71.9 (67.38, 76.02)	68.62 (63.75, 73.11)	68.59 (64.04, 72.81)	67.98 (63.26, 72.36)
≥65	31.22 (28.45, 34.14)	29.65 (25.85, 33.76)	31.99 (27.20, 37.18)	29.87 (25.85, 34.22)	27.32 (23.42, 31.61)	30.66 (26.56, 35.08)	28.1 (23.98, 32.62)	31.38 (26.89, 36.25)	31.41 (27.19, 35.96)	32.02 (27.64, 36.74)
**Gender**										
Women	53.01 (48.59, 57.37)	53.74 (50.23, 57.21)	54.95 (50.81, 59.03)	51.02 (47.09, 54.94)	51.6 (49.15, 54.05)	54.43 (51.92, 56.92)	52.9 (48.06, 57.69)	51.72 (48.95, 54.49)	52.6 (48.35, 56.82)	52.83 (46.98, 58.59)
Men	46.99 (42.63, 51.41)	46.26 (42.79, 49.77)	45.05 (40.97, 49.19)	48.98 (45.06, 52.91)	48.4 (45.95, 50.85)	45.57 (43.08, 48.08)	47.1 (42.31, 51.94)	48.28 (45.51, 51.05)	47.4 (43.18, 51.65)	47.17 (41.41, 53.02)
**Race**										
Non-Hispanic white	75.19 (69.10, 80.42)	80.59 (74.38, 85.58)	79.66 (72.14, 85.56)	79.18 (72.92, 84.30)	75.95 (68.43, 82.15)	73.46 (66.41, 79.49)	73.74 (65.67, 80.48)	72.86 (66.15, 78.67)	70.72 (61.32, 78.64)	69.89 (63.23, 75.80)
Non-Hispanic Black	9.17 (6.46, 12.87)	7.89 (5.31, 11.56)	9.67 (6.62, 13.93)	10.06 (7.20, 13.89)	10.63 (6.81, 16.23)	10.59 (8.25, 13.49)	10.49 (6.32, 16.93)	10.51 (8.04, 13.63)	9.23 (6.57, 12.82)	9.8 (7.62, 12.53)
Others	15.64 (10.31, 23.01)	11.53 (6.43, 19.81)	10.67 (6.96, 16.02)	10.76 (7.79, 14.68)	13.42 (9.87, 17.98)	15.95 (10.67, 23.17)	15.77 (12.54, 19.64)	16.62 (12.17, 22.29)	20.04 (14.08, 27.72)	20.31 (15.64, 25.94)
**Body mass index**										
Non-overweight	29.28 (23.48, 35.84)	27.27 (24.11, 30.68)	25.95 (21.30, 31.22)	30.96 (26.49, 35.81)	27.1 (24.04, 30.40)	26.33 (23.00, 29.96)	28.73 (24.99, 32.79)	25.09 (22.86, 27.46)	25.4 (20.23, 31.37)	20.59 (16.71, 25.10)
Overweight	37.53 (33.45, 41.81)	41.25 (36.86, 45.79)	41.21 (36.19, 46.41)	33.84 (31.54, 36.22)	38.36 (33.86, 43.07)	35.99 (31.95, 40.23)	34.3 (28.79, 40.28)	37.29 (33.63, 41.10)	33.68 (30.33, 37.21)	31.65 (28.02, 35.52)
Obesity	33.19 (28.24, 38.53)	31.48 (26.14, 37.36)	32.84 (28.55, 37.44)	35.2 (30.08, 40.69)	34.54 (30.03, 39.34)	37.68 (33.92, 41.60)	36.97 (31.07, 43.28)	37.62 (33.86, 41.55)	40.92 (35.64, 46.42)	47.76 (44.24, 51.30)

^a^Nationally representative estimate of the non-pregnant U.S. population aged 45 years or more through the use of survey weights. Data are expressed as percentages (95% confidence interval).

During a median follow-up of 106.83 (interquartile range: 52–152) months, 2,369 (24.78%) deaths were observed due to all-cause mortality, including 653 (27.25%) deaths from CVD and 562 (23.46%) deaths from cancer.

### Trends in urine lead

**Figure 1:**
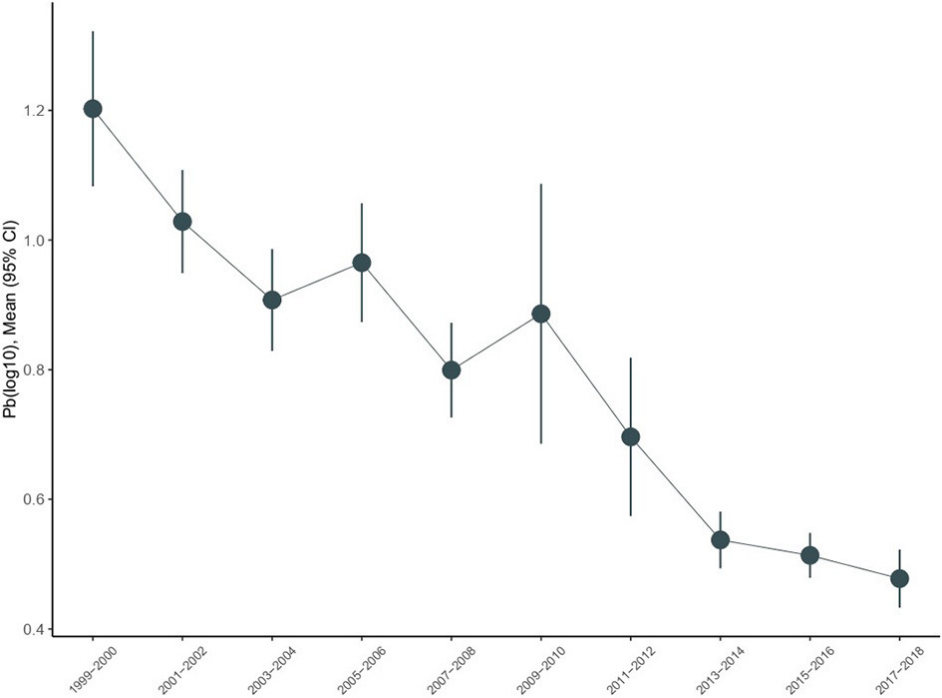
Temporal trends in urine lead in the National Health and Nutrition Examination Survey (NHANES), 1999–2018.

The trends of urine lead concentrations among US adults aged ≥45 years from 1999–2000 to 2017–2018 are shown in Figure 1. There was an obvious decline in urine lead concentrations from 1.203 μg/L (95% CI: 1.083–1.322) in 1999–2000 to 0.478 μg/L (95% CI: 0.433–0.523) in 2017–2018. Using the concentrations of urine lead in 1999–2000 as a reference, significant changes were observed for 2001–2002, 2003–2004, 2007–2008, and 2013–2014 (*P* = 0.018, 0.034, 0.006, and 0.017, respectively) (Table 2). Overall trends in urine lead concentration from 1999–2000 to 2017–2018 were statistically significant (*P* < 0.001).

**Table 2:** The magnitude of changes in mean lead concentrations in urine for subsequent NHANES cycles, using 1999–2000 as the reference^a^.

**Cycles**	**Mean**	**SE**	**Lower 95% CI**	**Upper 95% CI**	**Difference compared**	**P^b^**	**Overall P^c^ for trends**
1999–2000	1.202	0.061	1.083	1.322	Ref		< 0.001
2001–2002	1.028	0.0401	0.949	1.108	−0.174	0.018	
2003–2004	0.908	0.040	0.829	0.986	−0.121	0.034	
2005–2006	0.965	0.046	0.8739	1.057	0.058	0.348	
2007–2008	0.799	0.0370	0.7269	0.872	−0.166	0.006	
2009–2010	0.886	0.102	0.686	1.087	0.087	0.422	
2011–2012	0.696	0.062	0.5746	0.819	−0.190	0.112	
2013–2014	0.537	0.022	0.494	0.582	−0.159	0.017	
2015–2016	0.514	0.018	0.479	0.548	−0.0238	0.401	
2017–2018	0.478	0.023	0.433	0.523	−0.036	0.213	

NHANES, National Health and Nutrition Examination Survey; SE, standard error; 95% CI, 95% confidence interval; Ref, reference. ^a^Nationally representative estimate of the non-pregnant U.S. population aged 45 years or more through the use of survey weights. ^b^*P* value for the difference in mean lead concentrations in urine was calculated using the linear combinations of parameters. ^c^*P* value for overall trends was calculated using the Mann–Kendall trend test.

The concentrations of urine lead upon stratification by sociodemographic variables are provided in Table 3. The declining trends were comparable in adults aged 45–65 years and ≥65 years. Based on gender, women had higher urine lead concentrations than men on average across all NHANES cycles. A steeper decline in concentrations was observed in women (from 1.55 to 0.56 relative to men, from 0.89 to 0.40 in μg/L), and the difference between urine lead concentrations in men and women narrowed from 1999–2000 to 2017–2018. Based on race and ethnicity, the average concentrations of urine lead were consistently higher among non-Hispanic Black adults (from 1.55 to 0.60 μg/L) than among non-Hispanic white adults (from 1.14 to 0.46 μg/L). Based on the BMI category, differences in urine lead were not obvious for underweight or normal weight, overweight, and obese adults.

**Table 3:** Mean concentrations of urine lead across different subgroups in the National Health and Nutrition Examination Survey (NHANES), 1999–2018^a^.

**Subgroups**	**1999–2000**	**2001–2002**	**2003–2004**	**2005–2006**	**2007–2008**	**2009–2010**	**2011–2012**	**2013–2014**	**2015–2016**	**2017–2018**	***P* for trends^b^**
**Age, years**											
< 65	1.19 (1.07, 1.3)	1.03 (0.94, 1.11)	0.9 (0.8, 1)	0.93 (0.8, 1.06)	0.8 (0.71, 0.89)	0.93 (0.64, 1.23)	0.64 (0.53, 0.75)	0.51 (0.44, 0.58)	0.46 (0.42, 0.51)	0.46 (0.4, 0.52)	0.001
≥65	1.24 (1.02, 1.45)	1.03 (0.92, 1.14)	0.93 (0.81, 1.05)	1.04 (0.94, 1.14)	0.8 (0.74, 0.87)	0.79 (0.72, 0.86)	0.84 (0.63, 1.05)	0.59 (0.47, 0.71)	0.62 (0.56, 0.68)	0.52 (0.47, 0.57)	0.002
**Gender**											
Women	1.55 (1.39, 1.71)	1.29 (1.14, 1.44)	1.13 (1.02, 1.25)	1.14 (0.99, 1.28)	0.96 (0.83, 1.09)	1.13 (0.7, 1.56)	0.79 (0.57, 1)	0.66 (0.58, 0.75)	0.56 (0.51, 0.61)	0.56 (0.48, 0.64)	< 0.001
Men	0.89 (0.78, 1.0)	0.81 (0.73, 0.88)	0.72 (0.64, 0.8)	0.8 (0.68, 0.92)	0.64 (0.58, 0.71)	0.68 (0.6, 0.77)	0.62 (0.43, 0.8)	0.42 (0.38, 0.46)	0.47 (0.43, 0.52)	0.4 (0.34, 0.47)	< 0.001
**Race**											
Non-Hispanic white	1.14 (1, 1.28)	0.98 (0.89, 1.07)	0.87 (0.8, 0.94)	0.95 (0.83, 1.06)	0.75 (0.67, 0.83)	0.73 (0.67, 0.79)	0.69 (0.53, 0.85)	0.53 (0.48, 0.58)	0.5 (0.46, 0.54)	0.46 (0.4, 0.52)	< 0.001
Non-Hispanic Black	1.55 (1.21, 1.88)	1.46 (1.17, 1.75)	1.19 (0.99, 1.39)	1.05 (0.89, 1.21)	0.97 (0.76, 1.18)	1 (0.8, 1.2)	0.81 (0.57, 1.06)	0.61 (0.55, 0.67)	0.57 (0.49, 0.65)	0.6 (0.54, 0.66)	< 0.001
Others	1.31 (0.97, 1.64)	1.09 (0.91, 1.27)	0.91 (0.76, 1.06)	1.01 (0.81, 1.21)	0.95 (0.81, 1.09)	1.54 (0.38, 2.69)	0.65 (0.57, 0.73)	0.53 (0.42, 0.64)	0.53 (0.46, 0.59)	0.47 (0.4, 0.54)	0.009
**Body mass index**											
Non-overweight	1.08 (0.99, 1.17)	0.99 (0.81, 1.18)	0.99 (0.8, 1.18)	0.9 (0.77, 1.03)	0.88 (0.7, 1.07)	1.17 (0.49, 1.84)	0.75 (0.51, 0.99)	0.52 (0.41, 0.62)	0.53 (0.44, 0.62)	0.47 (0.38, 0.56)	0.005
Overweight	1.34 (1.02, 1.66)	1.11 (1.01, 1.21)	0.9 (0.77, 1.02)	1.0 (0.84, 1.16)	0.75 (0.66, 0.84)	0.87 (0.72, 1.02)	0.69 (0.43, 0.96)	0.54 (0.42, 0.67)	0.56 (0.48, 0.64)	0.47 (0.41, 0.54)	< 0.001
Obesity	1.16 (0.99, 1.34)	0.96 (0.86, 1.05)	0.86 (0.77, 0.95)	1.01 (0.89, 1.13)	0.79 (0.74, 0.85)	0.71 (0.65, 0.77)	0.67 (0.51, 0.82)	0.54 (0.45, 0.63)	0.47 (0.41, 0.52)	0.48 (0.41, 0.56)	< 0.001

^a^Nationally representative estimate of the non-pregnant U.S. population aged 45 years or more, obtained through the use of survey weights. Data are expressed as the mean (95% confidence interval). ^b^*P* value for overall trends was calculated using the Mann–Kendall trend test.

### Overall association between urine lead and mortality

Table 4 presents the risk estimates for urine lead that are associated with all-cause mortality, CVD-specific mortality, and cancer-specific mortality. Covariates were adjusted in a graded manner. Referring to the first tertile of urine lead concentrations, the risk magnitude for all-cause mortality was statistically significant and increased linearly before (*P* for trends < 0.001) and after adjustment (0.026 for the partially adjusted model and 0.020 for the fully adjusted model), and significance was attained for the comparison of the third tertile vs. the first tertile after full adjustment (HR = 1.17, 95% CI: 1.01 to 1.35). For continuous urine lead, the risk for all-cause mortality was statistically significant, irrespective of covariate adjustment (HR = 1.32, 1.18, and 1.19, 95% CIs: 1.15 to 1.51, 1.01 to 1.39, and 1.00 to 1.40 for the unadjusted, partially adjusted, and fully adjusted models, respectively).

**Table 4:** Association of urine blood concentrations in tertiles and on a continuous scale with all-cause mortality and disease-specific mortality^a^.

**Outcomes**	**Case/N**	**Model 1**	**Model 2**	**Model 3**
**All-cause mortality**				
T1	1809230/13279001	Ref	Ref	Ref
T2	2000044/10385160	1.17 (1.02, 1.35)	1.07 (0.92, 1.24)	1.06 (0.90, 1.24)
T3	2508509/9552418	1.28 (1.14, 1.45)	1.16 (1.01, 1.33)	1.17 (1.01, 1.35)
*P* for trends		< 0.001	0.026	0.020
Continuous (log10-transformed)		1.32 (1.15, 1.51)	1.18 (1.01, 1.39)	1.19 (1.00, 1.40)
**CVD-specific mortality**				
T1	12836678/13279001	Ref	Ref	Ref
T2	9796233/10385160	1.42 (1.09, 1.84)	1.27 (0.97, 1.66)	1.26 (0.95, 1.68)
T3	8959463/9552418	1.26 (0.97, 1.62)	1.12 (0.86, 1.46)	1.11 (0.84, 1.46)
*P* for trends		1.14 (0.93, 1.40)	1.04 (0.83, 1.31)	1.03 (0.81, 1.31)
Continuous (log10-transformed)		1.27 (0.97, 1.65)	1.09 (0.80, 1.48)	1.17 (0.78, 1.47)
**Cancer-specific mortality**				
T1	12839588/13279001	Ref	Ref	Ref
T2	9898531/10385160	1.19 (0.89, 1.60)	1.07 (0.79, 1.46)	1.03 (0.75, 1.42)
T3	8865655/9552418	1.51 (1.16, 1.95)	1.26 (0.96, 1.65)	1.26 (0.94, 1.67)
*P* for trends		1.43 (1.15, 1.79)	1.23 (0.98, 1.54)	1.25 (0.98, 1.58)
Continuous (log10-transformed)		1.60 (1.19, 2.15)	1.30 (0.94, 1.80)	1.32 (0.94, 1.86)

CVD, cardiovascular disease; Ref, reference. ^a^Nationally representative estimate of the non-pregnant US population aged 45 years or more through the use of survey weights. Data are expressed as the hazard ratio (95% confidence interval). For urine lead on a categorical scale, the first tertile was treated as the reference group. Model 1: no covariate was adjusted. Model 2: gender and age were adjusted. Model 3: gender, age, race/ethnicity, and BMI were adjusted.

Regarding the association between CVD-specific and cancer-specific mortality, there was no sign of statistical significance for urine lead on either the categorical or continuous variables after adjusting for covariates.

### Subsidiary association between urine lead and mortality

**Figure 2:**
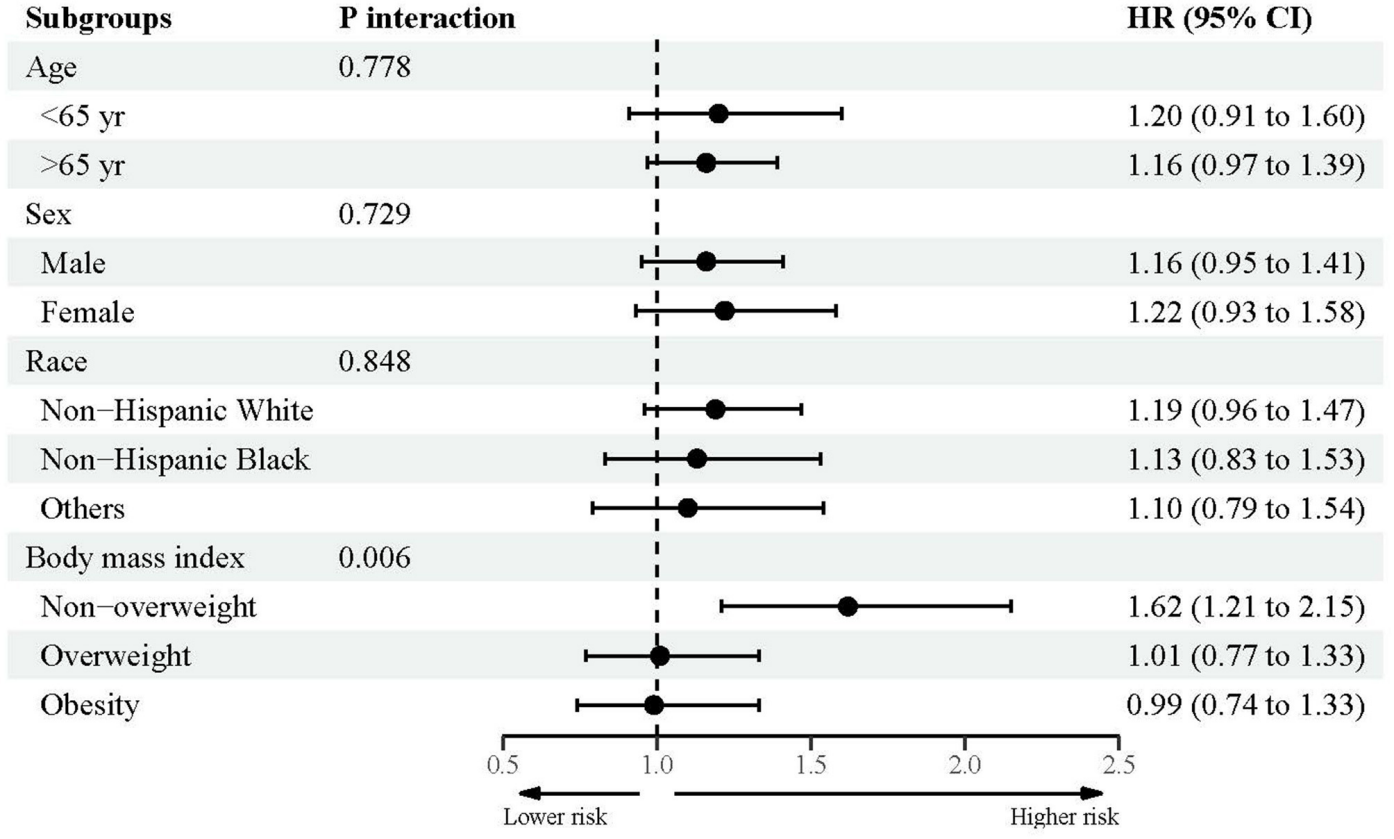
Subgroup analyses for the association between urine lead and all-cause mortality.

**Figure 3:**
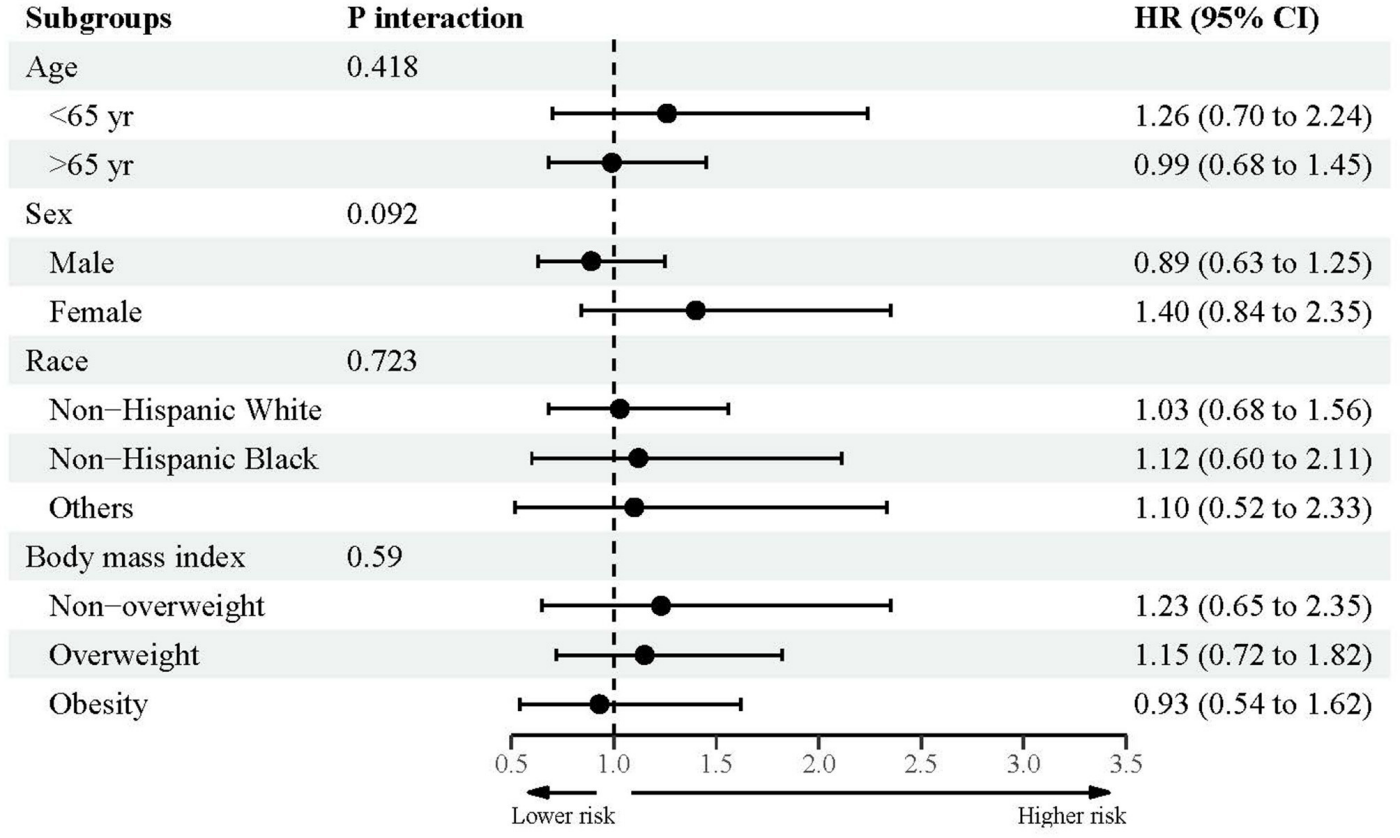
Subgroup analyses for the association between urine lead and cardiovascular disease-specific mortality.

**Figure 4:**
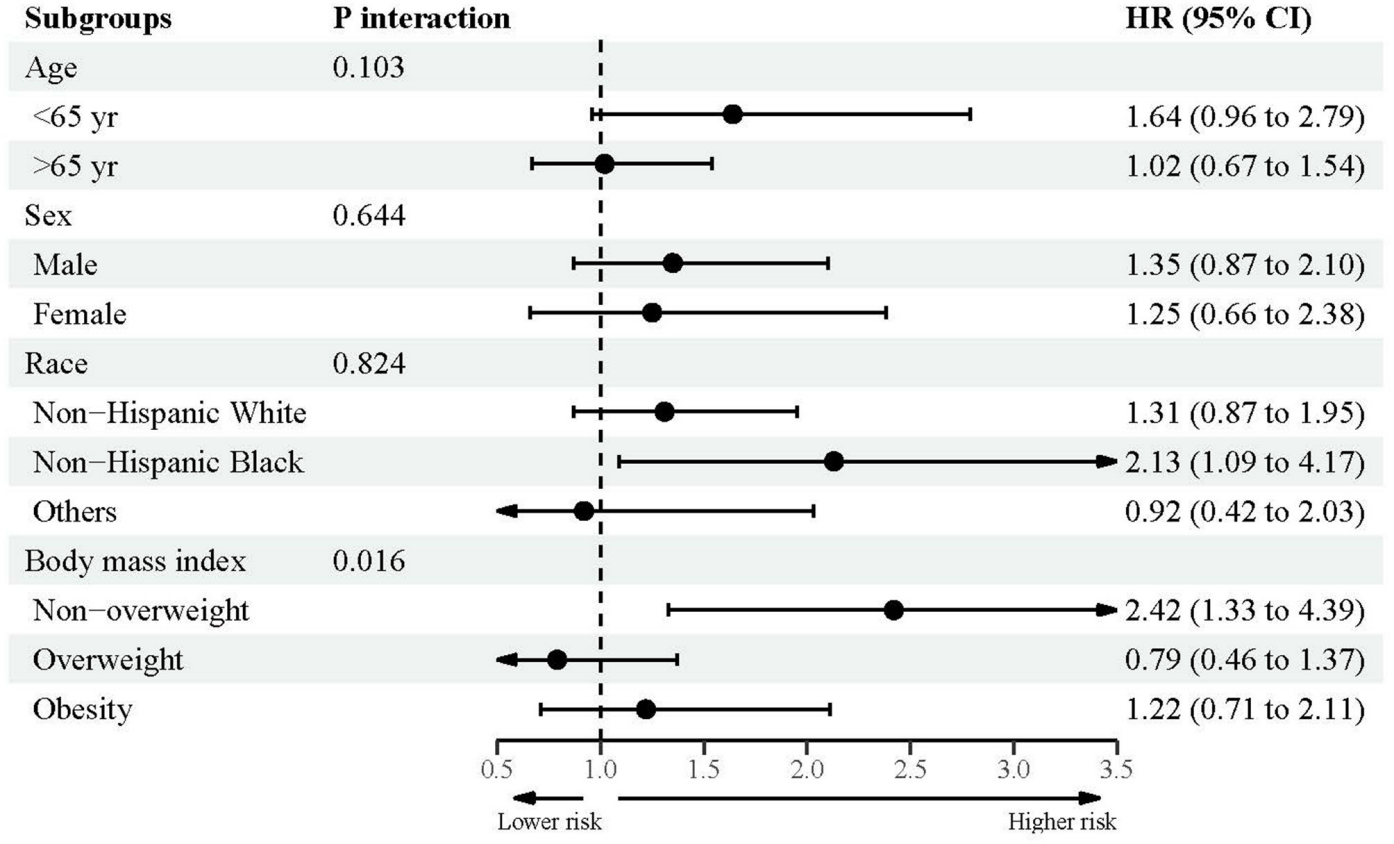
Subgroup analyses for the association between urine lead and cancer-specific mortality.

Figures 2–4 showed the subsidiary association between urine lead and all-cause and disease-specific mortality. The fully-adjusted association of urine lead with all-cause mortality was reinforced in underweight or normal-weight adults and reached significance, with per unit increment in log-transformed urine lead corresponding to 62% increased mortality risk (HR = 1.62, 95% CI: 1.21 to 2.15) (Figure 2). There was no observable significance for CVD-specific mortality across all subgroups (Figure 3). By contrast, adults of non-Hispanic Black descent and underweight or normal-weight adults were 2.13 (95% CI: 1.09 to 4.17) and 2.42 (95% CI: 1.33 to 4.39) times more likely to die from cancer, respectively (Figure 4).

### Dose–response relation between urine lead and mortality

Supplementary Figures S1–S3 displayed the dose–response relation between urine lead and all-cause and disease-specific mortality. The risk for all-cause mortality increased sharply with the increase of log-transformed urine lead from 0 to 1.5 μg/L and then remained stable with increasing lead concentrations (*P* for overall and non-linear tests: 0.011 and 0.019, respectively) (Supplementary Figure S1). For CVD-specific mortality, no significance was reached for the risk estimates with a 95% CI spanning the unity (*P* for overall and non-linear tests: 0.477 and 0.331, respectively) (Supplementary Figure S2). Regarding cancer-specific mortality, there was a steep increase in risk magnitude for an increase in log-transformed urine lead from 0 to 1.5 μg/L, and then the risk increased steadily with the increase in urine lead concentrations (*P* for overall and non-linear tests: 0.005 and 0.013, respectively) (Supplementary Figure S3).

## Discussion

This study aimed to describe the trends of urine lead among US adults aged ≥45 years over nearly two decades and to explore its association with all-cause, CVD-specific, and cancer-specific mortality. Overall, urine lead exhibited a declining trend from 1999–2000 to 2017–2018 in the United States. It is worth noting that high urine lead was a significant and independent risk factor for all-cause mortality, especially among underweight or normal-weight adults, and for cancer-specific mortality among non-Hispanic Black or underweight or normal-weight adults. To our knowledge, this is the first study to date to investigate the trends of urine lead and their association with mortality in the United States.

The trends in lead exposure have been widely evaluated worldwide. For instance, a recent Global Burden of Disease study in 204 countries and territories from 1990 to 2019 documented that lead exposure posed a significant disease burden, most of which occurred in men and the elderly population (16). Analysis of US pregnant women based on the NHANES dataset indicated a U-shaped distribution of blood lead concentrations from 2001–2002 to 2017–2018, with a nadir in 2013–2014 (17). Another NHANES analysis also supported the U-shaped pattern for blood lead concentrations from 1999–2000 to 2015–2016 in the general US population (18). By contrast, in this study, when focusing on urine blood concentrations, we observed a declining trend from 1999–2000 (1.203 μg/dL) to 2017–2018 (0.478 μg/dL) in US adults aged ≥45 years. Moreover, we interestingly observed that urine lead concentrations were, on average, higher in women than in men, with the gap narrowing over time, which was consistent with the findings of prior studies (19, 20). This declining trend can, at least in part, reflect the effective preventive strategies to lower disease the burden associated with lead exposure, such as tobacco control, air pollution reduction, hazardous-waste remediation, renovation of drinking water infrastructures, and banning lead in gasoline (1, 21, 22).

Another important finding of the present study was the association between urine lead concentrations and the significant risk of all-cause mortality in US adults aged ≥45 years. Growing evidence in the literature has supported that lead accumulation in the body is associated with a significant risk of mortality attributable to cardiovascular disease, cancer, and all causes (5, 6, 23–30). Differing from the results of prior studies, we only identified a significant association between urine lead on both categorical and continuous variable scales and all-cause exposure while failing to reveal any hints of significance for CVD-specific and cancer-specific mortality. Our findings were less likely to be biased by confounding factors, as adjustment was performed in a graded manner, indicating the robustness of this study. The possible causes behind the non-significant association between urine lead and CVD or cancer-specific mortality might be attributable to the limited number of respondents dying of CVD or cancer, relative to all causes. Moreover, the resultant divergence between prior studies and the present study might be due to the testing of blood or urine samples. It is universally accepted that urine is a better biomarker source than blood, given its changing nature. Unlike the “hide-and-seek” game played in blood, urine is not homeostatic and is supposed to be dumped as waste (31). Considering the complexity of lead-induced toxicity, which may be mediated by the production of reactive oxygen species resulting in oxidative damage, inhibition of endothelial nitric oxide synthase (32), and consequent adverse effects on health (33), we agree that more longitudinal studies are warranted to confirm or refute the findings of the present study.

In addition to the overall explorations, we additionally undertook subsidiary analyses on urine lead and mortality according to pre-specified confounders in this study. It is worth noting a significant association between cancer-specific mortality and urine lead in adults of non-Hispanic Black descent or those underweight or normal weight. In support of this note, the study by Cheung recorded that non-Hispanic Black adults were prone to cancer-specific mortality relative to white adults (34), which might be due to the diverging genetic profiles across different racial or ethnic groups. For instance, lead exposure was found to mediate the association between *JAZF1* gene rs10486567 and prostate cancer in African–American men but not in white men (35). Hence, it is important to construct a database of mortality-risk profiles for each racial or ethnic group. Regarding weight status, there is observational evidence that higher to lower lead concentrations were significantly associated with a lower likelihood of being overweight relative to normal weight (36). In other words, the detrimental impact of body lead in overweight or obese individuals was lower than that in underweight or normal-weight individuals, which may, at least in part, account for the significant association between urine lead and cancer-specific mortality in underweight or normal-weight adults. Despite this significant finding, the final proof of causality between urine and mortality, particularly CVD-specific and cancer-specific mortality, still requires further study.

### Limitations

In addition to the obvious strengths of this study, including the involvement of nationally representative adults, the large-scale sample size, and the long-term follow-up period, some limitations merit consideration. First, from 1999–2000 to 2017–2018, the response rate of the NHANES decreased from 76.62% to 48.24%, which made non-response bias an open question. Second, the lead that we relied on was from urine samples, and, therefore, the cumulative chronic or long-term exposure cannot be accounted for as a time-varying confounder in this study. In fact, the skeleton is a repository for 95% of the lead absorbed from deteriorated household paints, lead-contained water and food, and crystal or ceramic containers, and it can serve as an endogenous source for many years after exposure. Third, because of the declining trends in urine lead concentrations over nearly two decades, it is unclear whether the observed increased risk of mortality was due to lead exposure at baseline or lead mobilization from the skeleton. Fourth, only two specific causes of death were considered in this study, and due to the limited number of mortality data, it is too early to interrogate other causes such as kidney failure. Finally, although some sociodemographic variables were adjusted or stratified, there are still some unaccounted residual factors that might confound or mediate the association between urine lead and mortality.

## Conclusion

Despite these limitations, we, for the first time, described the declining trends in urine lead concentrations in US adults aged ≥45 years. Importantly, we found that urine lead was a promising marker that can help predict all-cause mortality. For practical reasons, effective preventive strategies to identify adults with high levels of lead exposure and accumulation, especially underweight or normal-weight adults, are imperative to improve population health and reduce healthcare costs.

## Data availability statement

The original contributions presented in the study are included in the article/Supplementary material, further inquiries can be directed to the corresponding author.

## Ethics statement

The studies involving humans were approved by National Center for Health Statistics Ethics Review Board. The studies were conducted in accordance with the local legislation and institutional requirements. Written informed consent for participation in this study was provided by the participants' legal guardians/next of kin.

## Author contributions

QW: Data curation, Methodology, Software, Writing—original draft. JW: Conceptualization, Formal analysis, Resources, Writing—original draft. XD: Supervision, Writing—review & editing. WN: Methodology, Software, Supervision, Validation, Writing—review & editing.
