# Supplementary material for: Trends in urine lead and associated mortality in US adults: NHANES 1999–2018
Source: Front Nutr. 2024 May 30;11:1411206. doi: 10.3389/fnut.2024.1411206 (PMC11169937; doi:10.3389/fnut.2024.1411206)

Figure S1. The dose-response relationship between urine lead and all-cause mortality.

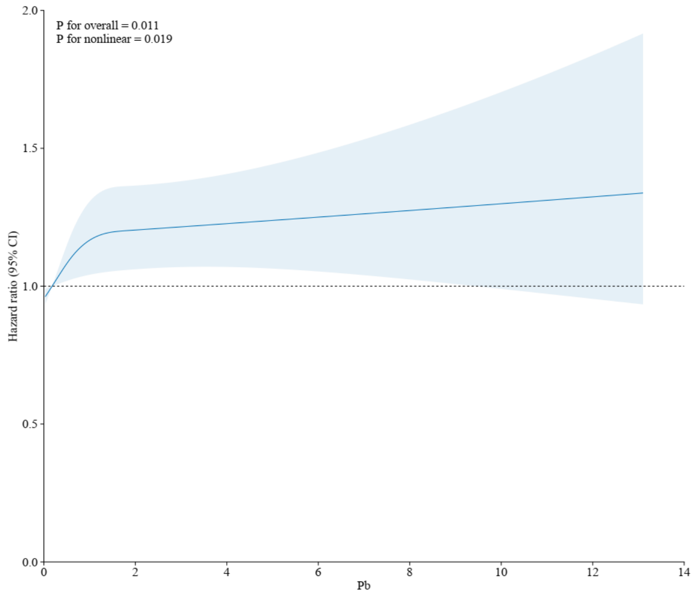

Figure S2. The dose-response relationship between urine lead and CVD-specific mortality.

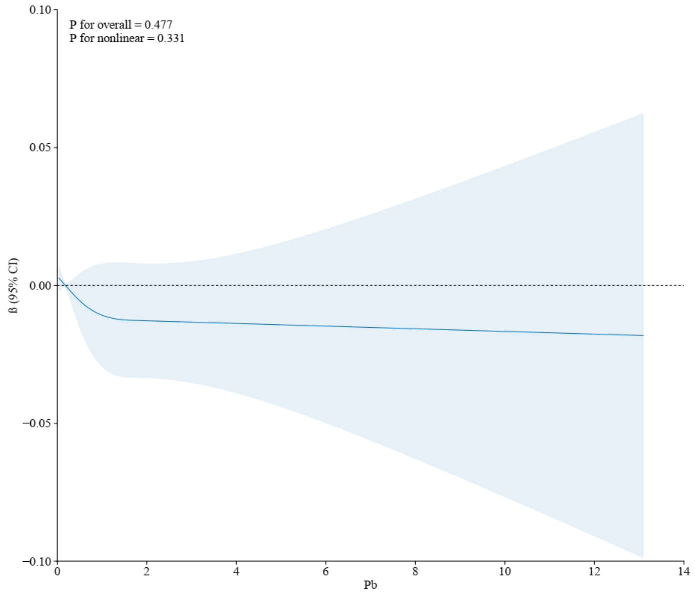

Figure S3. The dose-response relationship between urine lead and cancer-specific mortality.

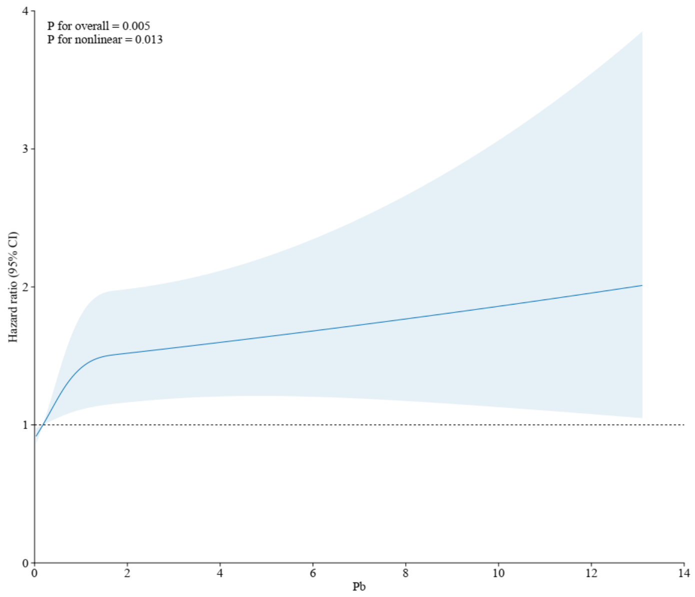

Supplement: Supplementary file 1 [file Image_1.pdf]
